# Placebo From an Enactive Perspective

**DOI:** 10.3389/fpsyg.2021.660118

**Published:** 2021-06-02

**Authors:** Iñigo R. Arandia, Ezequiel A. Di Paolo

**Affiliations:** ^1^IAS-Research Center for Life, Mind and Society, University of the Basque Country, Leioa, Spain; ^2^ISAAC Lab, Aragón Institute of Engineering Research, University of Zaragoza, Zaragoza, Spain; ^3^Ikerbasque-Basque Foundation for Science, Bilbao, Spain; ^4^Center for Computational Neuroscience and Robotics, University of Sussex, Brighton, United Kingdom

**Keywords:** enaction, embodiment, meaning response, agency, participatory sense-making, Gilbert Simondon, placebo & nocebo effects

## Abstract

Due to their complexity and variability, placebo effects remain controversial. We suggest this is also due to a set of problematic assumptions (dualism, reductionism, individualism, passivity). We critically assess current explanations and empirical evidence and propose an alternative theoretical framework—the enactive approach to life and mind—based on recent developments in embodied cognitive science. We review core enactive concepts such as autonomy, agency, and sense-making. Following these ideas, we propose a move from binary distinctions (e.g., conscious vs. non-conscious) to the more workable categories of reflective and pre-reflective activity. We introduce an ontology of individuation, following the work of Gilbert Simondon, that allow us to see placebo interventions not as originating causal chains, but as modulators and triggers in the regulation of tensions between ongoing embodied and interpersonal processes. We describe these interrelated processes involving looping effects through three intertwined dimensions of embodiment: organic, sensorimotor, and intersubjective. Finally, we defend the need to investigate therapeutic interactions in terms of participatory sense-making, going beyond the identification of individual social traits (e.g., empathy, trust) that contribute to placebo effects. We discuss resonances and differences between the enactive proposal, popular explanations such as expectations and conditioning, and other approaches based on meaning responses and phenomenological/ecological ideas.

## Introduction

Placebo effects have been and continue to be a source of controversy. There are disagreements about how to define them (Thompson et al., 2009; Moerman, 2013; Howick, 2017) and even doubts about their existence (Hróbjartsson and Gøtzsche, 2001, 2004). A significant body of evidence shows that robust and consistent bodily responses can follow medical interventions and that these responses are not caused directly by those specific interventions. They cannot be explained away by the natural evolution of the condition, regression to the mean, reporting bias, or observer effects. These responses depend on non-specific or incidental factors. They happen in a variety of conditions—e.g., pain, depression, anxiety, Parkinson's disease, sensorimotor limitations, addictions and behavioral disorders, immune and endocrine conditions, somatoform disorders—according to both subjective and objective measures. They follow different procedures—e.g., administration of pills, injections, ointments, technological interventions such as Deep Brain Stimulation in Parkinson's Disease or transcranial magnetic stimulation in other conditions, surgery, hypnosis, or through the therapeutic relationship itself. They occur both in clinical settings as well as in healthy subjects (Finniss et al., 2010; Ashar et al., 2017; Jensen, 2018). Controversies concerning placebo phenomena arise in part from the fact that these are group rather than individual effects. When looking at a particular patient, placebo effects are unpredictable and hard to reproduce. From being considered a confound to be eliminated, understanding placebo effects has become an important challenge for clinicians (Evers et al., 2018) and a source of ethical concern (Alfano, 2015; Hardman et al., 2019).

It is generally accepted that placebo interventions exert psychosocial rather than direct physiological influences on patients. Explanations are diverse but they all attempt to bridge a gap between psychology and physiology, looking at how both kinds of processes combine through the effects of expectations (e.g., Atlas and Wager, 2012), conditioning, and other forms of learning (e.g., Montgomery and Kirsch, 1997; Colloca and Benedetti, 2009), meaning responses (e.g., Moerman and Jonas, 2002), rituals (e.g., Kaptchuk, 2011), emotional modulations (e.g., Price et al., 2008), and interpersonal factors (e.g., Kaptchuk et al., 2008). These explanations are not mutually exclusive.

Despite this variety, several assumptions seem to be commonly accepted when carrying out empirical studies and framing explanations of placebo. For instance, assumptions about the relationship between psychology and physiology, between lived experience and health, between active and passive roles in the patient-practitioner encounter, and between social and individual factors. In questioning traditional assumptions, some authors have directed their attention to the roles of embodied personal and social experience (Frenkel, 2008; Thompson et al., 2009; Ongaro and Ward, 2017). Inspired by these ideas and following recent advances in embodied cognitive science, our main objective is to articulate an interpretation of placebo phenomena based on the enactive approach to life and mind (Varela et al., 1991; Thompson, 2007; McGann et al., 2013; Di Paolo and Thompson, 2014; Di Paolo et al., 2017, 2018; Fuchs, 2017; Gallagher, 2017), and examine the purchase enactive concepts may have for approaching placebo effects from a non-dualistic point of view. This perspective is promising and deserves to be introduced to the scientific community working on placebo research precisely because it overcomes traditional explanatory gaps between physiological, psychological, and social processes.

Our paper is organized as follows. First, we provide a brief overview of popular explanations of placebo effects and their associated assumptions. Then, we offer a general description of the enactive approach, linking key enactive ideas with existing explanations on placebo. We provide a detailed description of the enactive concept of dimensions of embodiment: organic/physiological, sensorimotor/psychological, and intersubjective/social. Following the philosophy of individuation developed by Simondon (2020), we extend the explanation of enacted bodies to include concepts of tension, metastability, preindividuality, and individuation. These concepts allow us to develop our relational-processual interpretation of placebo phenomena. Once our view of bodies and placebo effects is presented, we examine the sensorimotor/psychological and the intersubjective realms, investigating how they can influence the physiological dimension. We focus on sensorimotor agency, introducing the dynamic interplay between pre-reflective and reflective activity to replace the limiting dichotomic distinction between conscious and non-conscious processes. Then, we address the intersubjective domain by studying the concept of participatory sense-making in the context of the therapeutic encounter and other interactions. After describing how the physiological, the sensorimotor, and the intersubjective realms interact in placebo phenomena, we present some remarks on enactive conceptions of health. In the next section, we summarize our contributions to placebo research and offer suggestions to investigate enactive ideas empirically. Finally, we compare our approach with other explanations of placebo, discuss the limitations of our work and possible future developments, and conclude with thoughts related to the epistemic origin of placebo effects.

## Assumptions Behind Classical Explanations of Placebo

The most widespread account of placebo effects involves expectations, that is, a purely psychological construct that mediates between intervention and placebo effects (Kirsch, 1985, 1997, 2018). Positive expectations toward the intervention are postulated as causes for the positive effect of a placebo treatment. Expectations are not unitary entities. Kirsch separates stimulus and response expectancies. Stimulus expectancy is defined as the anticipation of external events that can alter perception (e.g., of painful stimuli), and response expectancy corresponds to predictions of non-volitional bodily responses. Expectations usually involve a combination of diverse processes involving the interaction between brain, body, and environment. Response expectancies tend to be “stronger, more stable, and more resistant to extinction” (Kirsch, 2018, p. 83), and also more relevant to explain placebo effects, as they are self-confirming. Although stimulus and response expectancies can be experimentally disentangled (Schenk et al., 2017), they usually overlap. Expectations have also been distinguished according to the level of reflectivity involved (Geers et al., 2019). Explanations based on expectations have received renewed interest within the popular framework of predictive processing and the free energy principle (Büchel et al., 2014; Ongaro and Kaptchuk, 2019). According to this framework, deviations from expectations update an internal model of the world that generates predictions about future interactions and events. By predicting bodily responses, expectations are hypothesized to elicit and modulate perceptions and induce changes through top-down mechanisms (Geuter et al., 2017).

Expectations, however, do not lead to individual placebo effects in a reliable manner. This could be because diverse psychological processes can also contribute to placebo phenomena: e.g., non-conscious goals and motivational factors (Geers et al., 2005), personality traits (Corsi and Colloca, 2017), hopes (Eaves et al., 2016), attention, emotional modulations, symptom attribution, anticipation (Geers and Miller, 2014; Horing et al., 2014).

Some experiments are better accounted for by conditioning rather than expectations, that is, a learning procedure that by pairing initially ineffective stimuli with effective ones, makes the former elicit “specific” responses associated with the latter (e.g., Benedetti et al., 1999). According to this view, previous experiences with clinicians, institutions (e.g., hospitals), or with certain treatments could be responsible for placebo effects. Both conscious and non-conscious learning procedures can induce or modulate bodily responses. Some researchers rely on non-conscious (implicit) expectations (e.g., Geers et al., 2019) to explain placebo effects elicited by purely non-conscious procedures (Jensen et al., 2012, 2015; Babel et al., 2017). The problem is that if conscious expectations are already highly fluctuating (e.g., Eaves et al., 2015) and difficult to grasp, measure, and manipulate, appealing to nonconscious expectations may not offer any additional verifiable explanatory power. Conditioning and expectations are not mutually exclusive (e.g., Stewart-Williams and Podd, 2004; Jensen, 2018) and several proposals combine them (e.g., Kirsch et al., 2016; Zion and Crum, 2018). Zion and Crum (2018) expand the notion of expectations to that of “mindsets,” integrating implicit learning mechanisms. They assume additivity among different factors and linear hierarchical influences from the social to the psychological to the physiological, but neglect feedback mechanisms across these levels and multiple scales (e.g., van Orden et al., 2003; Anderson et al., 2012).

To extract the underlying assumptions behind these explanations, it helps to remind ourselves of how placebo effects tend to be defined in empirical contexts.

In biomedicine, the use of placebos plays a key role in the evaluation of efficacy through randomized control trials (RCTs). Placebo effects have been interpreted negatively as “outcomes that cannot be measured in RCTs” (Sullivan, 1993, p. 224; Frenkel, 2008). Although this does not imply that they are an artifact of this methodology, the widespread view on placebo effects and placebo research tends to be linked to the theoretical and methodological assumptions of RCTs. Paterson and Dieppe (2005) point out three of these assumptions. First, the diagnosis is performed before the intervention and it is kept constant throughout the trial. In this way, the dynamic nature of the subject's response is limited and complex interventions that demand short-term adaptations, like physiotherapy, acupuncture, or psychotherapies, are difficult to assess using RCTs. Second, incidental or non-specific factors are generic and not linked to any particular therapeutic theory. However, what is non-specific in one intervention, as the therapeutic relationship in a drug trial, may be specific in another (e.g., psychiatry). Many studies lack an appropriate methodology to tell apart specific and non-specific effects, hindering replicability (Peper and Harvey, 2017). For instance, the psychological changes associated with the “real” treatment could contribute to believing the treatment is working (even when it does not). Active placebos, interventions that mimic the bodily sensations of the “real” treatment (without its specific effects), have been proposed to control for this situation (Boot et al., 2013). Third, specific and non-specific factors are assumed distinct and additive. In artificial and controlled experimental conditions, and for short-term timescales, physiological and psychological variables may be sufficiently disentangled (Benedetti et al., 2019). However, the apparent independence of physiological and psychological variables cannot be easily generalized from these cases to longer timescales. Linear additivity among different factors therefore can be acceptable in particular experimental settings (Benedetti et al., 2019), but not in general (Kleijnen et al., 1994; Coleshill et al., 2018). The tendency to study isolated variables looking for linear causes, while useful in particular cases, is not sufficient to fully comprehend complex phenomena across scales.

In addition to the assumptions identified by Paterson and Dieppe (2005), we can point to other common assumptions in RCTs. The separation between specific (physiological) and non-specific (non-physiological) factors implies a dualistic stance (Kirmayer, 1988). In RCTs, the specific is the physical and measurable. The uncontrollable other (i.e., psychological, social, and contextual factors) is labeled as non-specific. A dualistic split is assumed between the object body and the lived body. The object body is the measurable physical body that biomedical methods can access to define diseases objectively. The lived body, in contrast, is related to subjective experience, to illness, and demands a first-person perspective to grasp it. The biomedical paradigm is centered on understanding physical bodies and treating diseases. The necessity to rely on subjective measures (e.g., in pain or depression) is interpreted as a methodological weakness, as in reporting bias (Hróbjartsson and Gøtzsche, 2004); something best avoided. The patient becomes an object to be known by the practitioner, the knower. As a consequence, there is a tendency to treat patients—in RCTs, placebo research, and in biomedicine in general—as passive machine-like bodies responding to treatments in lawful ways. Agency, the sense of agency, and the capacity to generate and alter personal narratives are therefore downplayed, even when it is known that they affect experience, behavior, and bodily responses (Kirmayer and Gómez-Carrillo, 2019). Furthermore, in line with the individualist tendency of the biomedical paradigm, the socio-cultural environment is assumed to be an external influence playing at most a contextual, modulatory role (although of increasingly acknowledged importance, see e.g., Wager and Atlas, 2015). All of these separations emerge from adopting a dualistic stance that ignores or downplays the underlying relations between the contrasting elements (specific/non-specific, physiology/psychology, physical body/lived body, knower/known, individual/sociocultural). They affect RCTs and placebo research in general. For instance, expectations are considered as mental representations separate from non-volitional bodily responses but somehow able to affect them. Dualism also underlies the dichotomy between conscious and non-conscious processes in explanations in terms of expectations and conditioning.

To summarize, the assumptions behind many explanations of placebo are (1) *reductionism and linearity*, the attempt to isolate variables and posit linear causal links, (2) *dualism* as revealed by the separation between specific and non-specific factors, between objective and subjective measures, and between physiology and psychology, (3) the relegation of lived *experience* to a secondary explanatory role, (4) a tendency to remediate dualism with *representationalism* i.e., conceiving cognition as the manipulation of mental representations mediating between the separable processes of perception and action, (5) the *passivity* of individuals undergoing procedures and the neglect of their agency, (6) an excessive *individualism* that assigns a mere contextual secondary role to participatory and sociocultural processes, and (7) the *limited temporality* of experiments that downplay the complex dynamic and historical nature of living bodies.

Given that these assumptions work more pervasively by not being out in the open—despite having been repeatedly criticized—it is not surprising that placebo effects remain such a source of controversy. These assumptions may be acceptable in pharmaceutical trials, but the limitations they impose on placebo explanations cannot be overlooked.

## The Enactive Approach: a Brief Overview

In this section, we briefly overview some basic enactive ideas and in the sections that follow, we focus on specific concepts that we deem of particular relevance for placebo research: dimensions of embodiment, agency, and participatory sense-making. This will enable our goal of evaluating what these ideas—as developed in the enactive approach—can contribute to placebo research.

The enactive perspective is an active strand in embodied cognitive science, part of what is known as the 4E approaches: embodied, embedded, extended, enactive (Newen et al., 2018)^1^. It offers a naturalistic approach to life and mind (Varela et al., 1991; Thompson, 2007; McGann et al., 2013; Di Paolo et al., 2017, 2018; Fuchs, 2017; Gallagher, 2017). The enactive approach is strongly influenced by phenomenology, pragmatism, dynamical systems theory, and organizational approaches in biology. One of its central premises is that of a *continuity between life and mind* that accepts the relative qualitative distinctions of different kinds of biological and mental phenomena while at the same time stressing their deep connections in a non-reductionist manner (Thompson, 2007; Di Paolo et al., 2017). A central enactive concept is that of *autonomy* (Varela, 1979; Di Paolo, 2005; Di Paolo and Thompson, 2014). Autonomy is a technical and operationally defined concept at the root of the enactive approach (that should not be confused with more general uses of the term). Autonomous systems, such as organisms, are defined in systemic terms as operationally closed and precarious networks of mutually enabling processes. They are materially self-constituting (self-producing and self-distinguishing). At the same time, they require active engagements with the external enabling relations on which they depend. A living unicellular organism is a paradigmatic example of autonomy (in this case, autopoiesis). It is a self-individuating material entity that emerges from a continuous and precarious process of self-production (of its organizational integrity) and self-distinction (from its environment). Its complex metabolic pathways, many of which are auto-catalytic, produce and repair a semipermeable membrane, which in turn contains and enables the metabolic reactions instantiating a network of operationally closed processes. Autonomous systems can exist at various scales, from metabolism and immune activity to nervous, sensorimotor, and social dynamics. And these multi-scale autonomous processes can influence, constrain, and enable each other in complex ways.

The material individuation of an organism is simultaneously the emergence and specification of an (organism-relative) environment, which arises, formally and materially, with a myriad of possibilities for meaningful interactions (Varela, 1997; Weber and Varela, 2002). Autonomy provides organisms with a perspective on the world, according to which events are meaningful insofar as they affect the continuation of their precarious processes of self-individuation. These meaningful relations are not ascribed externally but correspond to the organism itself. To operationalize this idea, enactivists speak of *adaptivity* (Di Paolo, 2005), a system-theoretical concept that allows us to explain how the organism can follow its vital norms (Thompson, 2007) by telling apart dangerous from safe situations, e.g., nutrient-poor from nutrient-rich environments. *Sense-making*, then, is defined as “the capacity of an autonomous system to adaptively regulate its operation and its relation with the environment depending on the virtual consequences for its own viability as a form of life” (Di Paolo et al., 2018, p. 33). This is a general concept that describes the structure of *all* mental phenomena, including their cognitive and affective aspects. It expresses the deep relation between the continued existence of an agent and its concrete situation as a relation of being attuned to, or *caring* for, what matters. The combination of sense-making, precarious autonomy, and adaptivity leads to a non-dualistic naturalization of the core aspects of the mind and underlies every process of perception, action, emotion, and cognition. They all share a basic existential/experiential structure of *concern* or *caring*. Based on these concepts, enactivists propose three requirements that are necessary for an organism to be considered an *agent*. (1) *self-individuation*: agents must actively differentiate themselves from their surroundings. (2) *interactional asymmetry*: agents must be capable of altering their coupling with the environment (not only being affected by it). (3) *normativity*: agents must follow the norms that emerge from their form of life (even if they can incorporate external norms). Agency marks the difference between an event that simply occurs and an act that is performed.

## Enactive Ideas and Existing Explanations of Placebo

Before we proceed to elaborate more specific potential contributions from the enactive approach to placebo research, we should mention that, broadly, enactive ideas already resonate with ecological, phenomenological, meaning- and person-centered explanations of placebo phenomena. These already existing parallels are worth a brief mention here.

The concepts of agency and sense-making resonate with meaning-response accounts of placebo phenomena (Moerman and Jonas, 2002; Goli, 2016). According to this viewpoint, it is not placebos as such that cause placebo effects. The stimulating effect of red-colored “inert” pills and the tranquilizing effect of blue ones are a direct consequence of the meaning they generate. Meaning responses explain why placebo effects are more pronounced with more invasive procedures (Meissner et al., 2013), the influence of awareness of a treatment (e.g., Colloca et al., 2004), and the need for active placebos (Boot et al., 2013) to control for meaning responses to bodily sensations following “real” interventions. A meaning response does not refer only to conscious semantic meaning (Kirmayer, 2003; Thompson et al., 2009). It is important not to interpret the meaning as another representationalist mediator linking the inner and the outer world (Taylor, 2006), but as a series of processes that can be triggered or modified by a placebo intervention. This is in agreement with the enactive understanding of meaning as the *activity* of a fully embodied autonomous agent, i.e., *sense-making*, rather than something “in the head.”

The enactive approach also resonates with phenomenological/ecological perspectives on placebo. Frenkel (2008) draws inspiration from the concept of affordances in ecological psychology, that is, the possibilities for action that the environment presents to the agent (e.g., a doorknob affords being turned to open a door). Frenkel proposes to move from meaning responses to affordances of healing, which he defines as solicitations of the environment that allow improving a health condition. They are procedures that allow responses that improve a particular situation. However, an affordance does not entail a response. A door that affords opening remains closed until someone acts on it. The action may even fail. Placebo interventions open a possibility for a response but do not actualize it. Moreover, this possibility is not given to conscious or reflective responses only but can include pre-reflective and/or non-conscious activity. In other words, sense-making applies to the whole active organism concerning its environment. Meaning hence is tightly linked to agency, embodied experience, history, and situation.

Ongaro and Ward (2017) also emphasize the role of affordances, which they link to a shift in attention from the figure (environment) to background (body) in pain or illness. While agreeing with this interpretation, we will elaborate a perspective in terms of the interplay between pre-reflective and reflective activity that includes attentional modulations but also habits, readiness, emotional dispositions, and action tendencies (see section “The Interplay between Reflective and Pre-Reflective Activity”). Ongaro and Ward (2017) also highlight the benefits of placebo research of adopting an enactive perspective because it is inherently affective and intersubjective. Emotions, in this view, are not private states but involve the whole embedded organism. Affectivity arises in meaningful relations with the environment and as dynamical configurations of bodily activity (Colombetti, 2014). Human sense-making is transversed by personal habits, social practices, language, and cultural narratives. If culture, cognition, and affectivity are part of interrelated systems, it should not surprise us to observe bodily responses following culturally meaningful events. Our goal is to expand on Ongaro and Ward's (2017) proposal, articulating and deploying enactive tools to further investigate the “web of dynamic relations between mind, body, and world, between affect and cognition, and between self and society” (ibid. p. 530).

In the following sections, we elaborate on this brief overview of enactive concepts by articulating the relations between dimensions of embodiment, reflective and pre-reflective aspects of agency, and participatory sense-making. We acknowledge the crucial role that the affective (Colombetti, 2014), the experiential and the existential (de Haan, 2020) dimensions play in the enactive approach and their relevance for placebo phenomena. While we touch on some of these aspects, due to space constraints, we will not elaborate on them in the present work.

## Living and Enacted Bodies

In this and the following sections, we expand and elaborate on some enactive concepts to offer more specific tools for building explanations of placebo effects.

Human bodies enact similar (though not identical) processes of autonomy, sense-making, and agency simultaneously, giving rise to the organic, sensorimotor, and intersubjective dimensions of embodied self-regulation (Thompson and Varela, 2001).

The organic dimension is characterized by metabolism, immune and hormonal regulation systems, and other physiological processes. The sensorimotor dimension corresponds to self-induced neural activity as well as perception-action loops, which are organized in plastic and dynamic networks of interrelated behavioral patterns or sensorimotor schemes (Di Paolo et al., 2017). As these schemes organize themselves into networks of structural and functional relations, the resulting sensorimotor repertoire (skills, powers, sensitivities) can also meet the requirements for agency (self-individuation, interactional asymmetry, and normativity). Sensorimotor agency is another form of autonomy, different from, but entwined with organic autonomy. Habits are an example of self-sustained sensorimotor relational patterns that ground a set of norms underdetermined by purely organic normativity (as shown by the difficulties in changing “bad” habits that negatively affect organic processes, e.g., addictions, Ramírez-Vizcaya and Froese, 2019). Finally, the intersubjective dimension describes the circular processes of generating and transforming meaning and constituting personhood through social interactions, language, and social norms.

**Figure 1:**
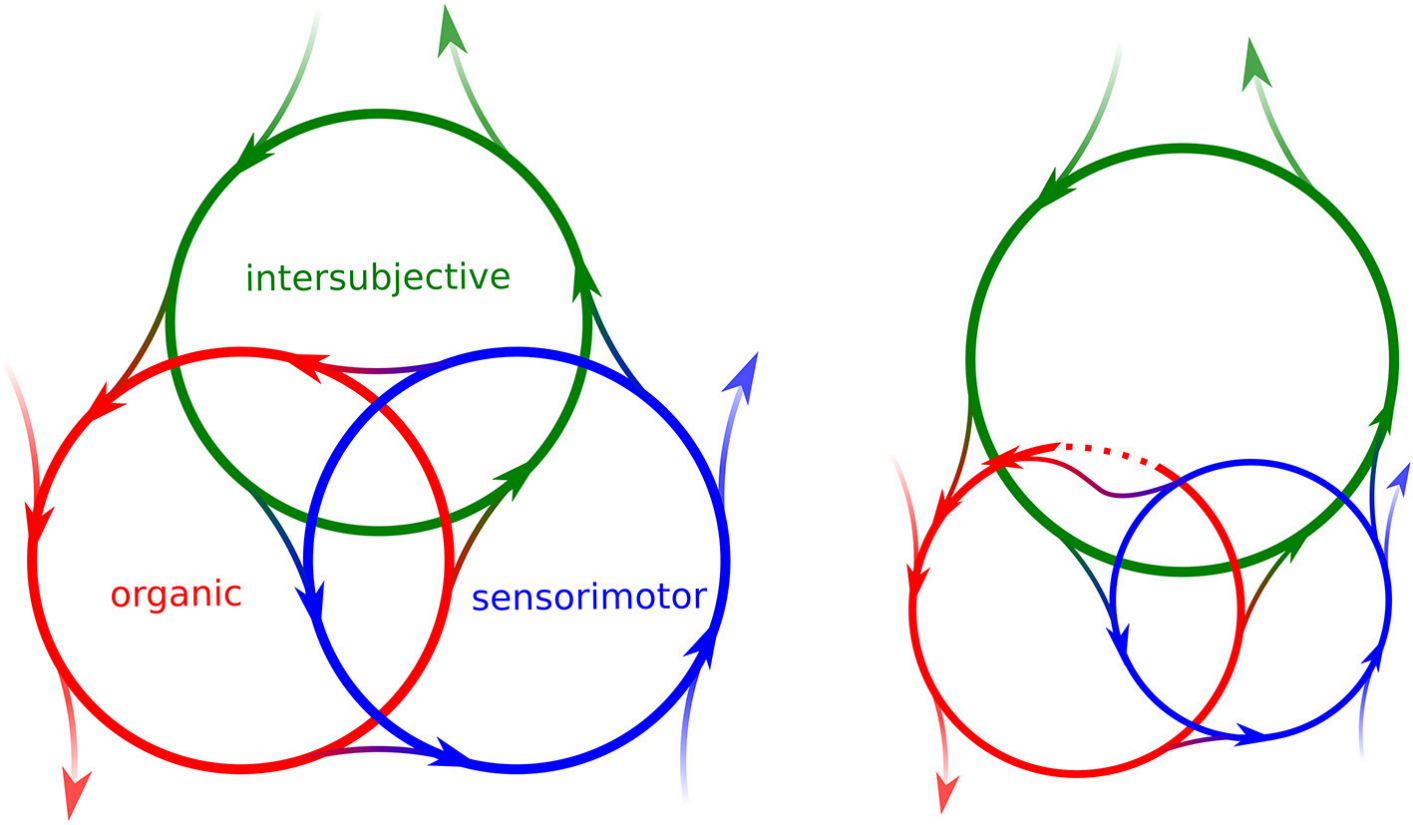
Dimensions of embodiment. Left: the organic (red), sensorimotor (blue), and intersubjective (green) dimensions of embodiment are composed of cycles or loops (circles), interrelated in a non-hierarchical way and deeply influencing each other (arrowed lines between circles). The regulation of each dimension occurs under precarious conditions, that is, in interaction with the environment that can enable, facilitate or constrain internal constitutive processes (lines directed outwards or toward a loop). Right: a disorder in an organic loop (broken red cycle), e.g., arterial disease, can limit sensorimotor capacities (reduced blue circle). A disruption in the organic cycle can begin to be compensated by the sensorimotor dimension (walking therapy), which is supported by the intersubjective loop (interaction with trainers).

It is important to note that each dimension can include several interrelated cycles or loops (Kirmayer and Gómez-Carrillo, 2019), which makes them more challenging to study than linear causal chains, but not impossible. Furthermore, the three dimensions are interdependent, dynamically intertwined in a non-hierarchical way constituting a precarious, autonomous, self-individuating, but often also self-contradictory human body (Figure 1). The interplay between these dimensions makes it hard to separate causes and effects. Stilwell and Harman (2019) discuss the limitations of splitting a non-decomposable phenomenon such as pain into biological, psychological, and social components. The interactions are not additive and, as a consequence, each dimension cannot be investigated in isolation (Di Paolo et al., 2018, ch. 5). Consider patients with arterial disease in the lower limbs undergoing walking therapy. Vascular obstruction reduces blood flow, generating pain and limiting walking capacity. “Walking therapy does not intervene in vessels. Yet it may lead to clinical improvement; that is, it may increase a patient's pain-free walking distance.” (Mol, 2002, p. 226). There is no single disease in the arteries, but an embodied experience of pain and practical limitations, that make walking therapy an appropriate treatment. Moreover, the attitude of the trainer deeply influences the process. The intersubjective dimension is fundamental for the sensorimotor activity to have a positive effect on a problem of organic origin. In turn, increasing pain-free walking distance also enhances the activity and social life, and alters personal narratives that impact future social behavior—another looping effect.

What is the role of the brain in this picture? The enactive approach moves away from neurocentric perspectives. Through enactive lenses, “the brain is conceived as a plastic system of open loops that are formed in the process of life and closed to full functional cycles in every interaction with the environment” (Fuchs, 2011, p. 196), a “mediating organ” that despite playing a crucial role in the meaningful interactions between body and environment, does not give rise to lived experience all by itself (Thompson and Cosmelli, 2011); despite contributing to mental processes, it does not create the mind; despite being crucial for reasoning, perceiving, and feeling, it does not think, perceive, or feel by itself. It is the whole body in interaction with its environment that does all of these things, and not any part of it. The enactive approach avoids mereological fallacies typically encountered in neuroscience (Bennett and Hacker, 2003).

Several perspectives on brain function are largely compatible with these enactive ideas (e.g., Kelso, 1995; Freeman, 2000; Varela et al., 2001; Anderson, 2010; Pessoa, 2013; Tognoli and Kelso, 2014; Raja, 2018). Cognition, then, is what happens in the coupling between the bodies of agents and their environment (and the affectivity that emerges in this engagement), and not what happens inside their heads. Perception and action are not two independently accessible processes dealing with inputs and outputs, respectively, but codependent facets of closed sensorimotor loops. They give rise to the sense of agency, a sense of being situated in a meaningful world.

## A Relational-Processual Conception of Placebo Effects

To better understand the enactive dimensions of embodiment, we introduce in this section some key ideas of Gilbert Simondon's (2020) philosophy of individuation^2^. This will contribute to formulating a relational-processual interpretation of placebo phenomena.

Over the last decade, Simondon's work has become increasingly influential on the development of enactive ideas (e.g., Stewart, 2010; Thompson, 2011; Di Paolo et al., 2018; Dereclenne, 2019; Di Paolo, 2020). In particular, Simondon's philosophy of individuation complements enactive concepts such as autonomy, agency, and sense-making by explicating the material and relational conditions that underlie different kinds of individuation, from physical to social. Simondon advances an ontology of becoming that puts the ongoing processes of individuation in a place of priority over the fully formed individual. This helps clarify the material conditions under which biological and cognitive “individuals” are inescapably subject to multiple tensions across different modes of individuation. Rather than being fully formed once and for all, the regulation and transformation of these tensions make up a body's ongoing becoming. In this way, Simondon's philosophy helps us make better sense of the different dimensions of embodiment by escaping the age-old dualism of matter and form (hylomorphism) that is prevalent in most current approaches in biology and psychology, including organizational theories of life such as the classical theory of autopoiesis (see DiFrisco, 2014; Di Paolo, 2018).

The entwinement and circularities within and between the dimensions of embodiment make tensions and conflict ubiquitous phenomena. Acknowledging this fact can lead to a major switch in how we approach placebo phenomena. By tension, in this context, we specifically mean the state of a system or process being subject to multiple regulatory demands simultaneously. There are tensions between sympathetic and parasympathetic nervous activity, between insulin and glucagon in glycemic regulation, and so on. An excess or a lack can lead to disease, and eventually, be fatal. Tensions also emerge in the sensorimotor dimension (e.g., between flexor and extensor muscles, between multiple affordances and intentions) and in the intersubjective one (e.g., between being oneself and submitting to the demands of an interactive encounter). And also between dimensions of embodiment through conflicting normativities, as in habits and addictions. From the enactive perspective, life is not a harmonious process where all the different parts necessarily cooperate for the common good, but the result of generating, releasing, and transforming tensions between processes, under the ever-present possibility of breakdown and death.

The work of Simondon (2020) provides tools to make sense of these different kinds of tension. He offers a processual ontology for understanding entities (including living organisms) as resulting from ongoing processes of individuation rather than as finished, ontologically complete beings. Without going into too much detail, the paradigmatic example of physical individuation is the formation of a crystal in an oversaturated solution. A perturbation (e.g., a seed) to the metastable liquid solution triggers a phase transition, releasing part of the chemical potential and leading to another (lower energy) metastable state. The perturbation is not responsible for crystallization, it is a *trigger*. The oversaturation of the solution and the tensions and potentialities it generates (what Simondon calls the *preindividual*) are essential for the perturbation to have any effect. We cannot understand the process of individuation just by examining the trigger. This idea, together with the notion of the preindividual, have sometimes been implied but never made explicit in previous enactive work.

In contrast with physical individuation that releases tension in one go when triggered by external stimuli, vital individuation involves an ongoing process of regenerating tension. Simondon understands living organisms as self-organized entities that actively renew tensions to keep themselves far from equilibrium (i.e., death), opening up new potentialities and becoming a source for future individuation processes. When tensions cannot be managed through organic processes alone, they can be regulated by the interdependent psychic and collective forms of individuation. Psychic individuation is related to the sensorimotor dimension of embodiment. Psychic individuation regulates tensions between internal and external processes and between individuated structures and preindividual potentialities (i.e., past and future) that give rise to affective states. It does so through processes described as actions, perceptions, emotions, memories, thoughts, and so on. Perception integrates different sensations, perhaps confusing or conflictive, into a meaningful whole, like the disparity between the two-dimensional images in each retina that is solved by individuating a perception of depth. Likewise, emotion makes different affects compatible along multiple axes (e.g., pain/pleasure, curiosity/boredom, risk/safety, hunger/satiety) to organize action. Simondon argues not only in favor of the continuity between life and mind but also between individuals and society. Psychic individuation requires participation in the collective. This does not mean that uninterrupted social interactions are necessary to carry out psychic individuation (e.g., to generate instances of perception and emotion), but that those processes depend on previous interactions and collective individuation (e.g., developing in a given socioeconomic condition, learning a certain language, acquiring a particular habitus). Through shared practices, social groups continuously generate collective individuations, such as institutions, values, prejudices, morals, science, art, and so on.

What this view suggests is that a placebo intervention should be understood as a *perturbation* that modifies the configuration of existing tensions and that may or may not trigger an individuation process. Placebo effects, then, are individuation processes in a configuration of pre-existing tensions that, in analogy with the case of crystallization, amplify local effects, transducing between micro- and macro-scales, between somatic and psychic activity, and induce new coherences through the propagation of internal resonances. Placebo effects are bodily responses that “solve” a certain problem or blockage and release or regulate accumulated tensions. But not all interventions trigger an individuation process leading to a placebo response. These responses depend on how the intervention triggers processes of sense-making and how they contribute to personal meaning. And this depends on a person's experiences and current context understood not just as static situational states (e.g., beliefs, expectations), but as ongoing processes of individuation. Such individuation processes are not exclusive of placebo interventions. They can be triggered, facilitated, or curtailed by therapeutic encounters and other meaningful interactions. They can contribute to a healing process (placebo) or trigger a cascade of processes that increase other tensions, leading to diverse symptoms, and hindering well-being (nocebo). Or they can fail to trigger any significant change at all.

With this conception of placebo, in the following sections, we explore how sensorimotor and intersubjective dimensions can affect the physiological dimension describing and applying the enactive concepts of agency and participatory sense-making.

## Agency

There is a tendency in placebo research to treat patients' bodies as passive, neglecting the role of the patient's agency. However, active patient involvement in decisions about treatment leads to more pronounced placebo effects (Geers et al., 2013). The effects of an intervention are often measured according to the evolution of isolated symptoms. By contrast, we argue that symptoms must be framed within the lived experience of the individual, her current practices, and her sociocultural context. Symptoms manifest the active nature of the body, as they result from attempts at regulating existing tensions. A patient's history, motivations, worries, habits, and current actions are not incidental or merely contextual factors in placebo phenomena.

In the case of patients with arterial disease mentioned earlier (Mol, 2002), pain-free walking distance is one measurable variable that contributes to (but does not determine) lifestyle limitations. Importantly, the success of walking therapy is not established by the state of the arteries. Factors like effort, motivation, worries, expectations, hopes, and the relationship with the trainer or therapist are relevant as well (Stilwell and Harman, 2017; Kinney et al., 2020).

Interestingly, trainers tend to hide their role in the effectiveness of walking therapy. “[T]he idea that the results of walking therapy are one's own achievement is a boost for a patient's self-confidence.” (Mol, 2002, p. 230). So, not only does agency as such matter (see also Collins et al., 2010), the sense of agency and perceived self-efficacy also play a key role in therapeutic settings (O'Leary, 1985). From an enactive perspective, studies manipulating perceived self-efficacy in therapeutic settings are continuous with placebo phenomena (Bootzin and Caspi, 2002; Thompson et al., 2009), because they involve bodily responses following concrete interventions that cannot be explained by the interventions themselves. They are related to misattributed action outcomes (i.e., altered perceived self-efficacy) instead of misattributed (or amplified) bodily sensations as in typical placebo responses (e.g., Weinberg et al., 1984).

As we have discussed, human bodies are not given, already individuated entities from which actions emerge but are co-defined and co-constituted by what they do in the world. Consequently, well-being is a result of the activity and situatedness of embodied agents. Breakdowns in well-being correspond to breakdowns in our possibilities as agents. In the phenomenological literature, pain and illness are interpreted as breakdowns interrupting activity and directing attention toward the body (Toombs, 1988; Colombetti, 2011). Similarly, modulating attention can contribute to placebo effects (Geers et al., 2006), by altering behavioral patterns, or displaying environmental affordances that facilitate healing (Frenkel, 2008; Ongaro and Ward, 2017). From this viewpoint, any intervention altering the perception of the environment (Trimmer et al., 2013, p. 13) will affect placebo phenomena. But it does so by impacting the network of interrelated behavioral patterns that constitute sensorimotor agency (Di Paolo et al., 2017). Therefore, one of the goals of placebo research, we suggest, should be to unveil the connections between meaningful interventions and sensorimotor repertoires—involving attention, habits, and everyday practices—, taking into account that they are not fixed, but plastic, dynamic, and sensitive to experience and social interactions.

## The Interplay Between Reflective and Pre-Reflective Activity

An obstacle to revealing the role played by agency in placebo phenomena lies in the fact that sensorimotor schemes are not always overtly activated but can still influence action, perception, and emotion (Dewey, 1922/1988). Habits, attitudes, and trait concepts (e.g., “being old”) can elicit behavioral changes (Hacking, 1999). The situation is usually dichotomized into conscious and non-conscious influences, a simplification that favors attention on conscious processes while consigning non-conscious processes to an empirically inaccessible shadow, a hidden variable open to unbridled speculation. We have already mentioned some of the limitations of the conscious/non-conscious divide. It is also dichotomous, leaving little room for theorizing about the relation between the two terms. It is methodologically problematic as non-conscious processes are often lumped together with non-intentional activity and any other factor whose influence is difficult to articulate. For this reason, it risks generating confusion and leads to problematic constructs, such as “non-conscious expectations” or, by contrast, focusing too much on only the conscious aspects of a meaning response. To overcome these limitations we consider the dynamic interplay between the pre-reflective and reflective sense of agency.

The difference between the pre-reflective and the reflective experiences of agency (Gallagher, 2012) follows the phenomenological distinction between pre-reflective and reflective forms of experience (Legrand, 2007; Gallagher and Zahavi, 2013). Both are forms of embodied intentionality. Briefly, the reflective aspect of the sense of agency is transitive (i.e., involving a subject-object distinction), explicit, and phenomenologically dominant, while the pre-reflective aspect is intransitive, often implicit, and phenomenologically recessive (i.e. noticeable when the flow of our actions is disrupted). Many approaches in placebo research focus on the reflective, communicable aspects of experience. However, in everyday actions (e.g., opening a door), in habitual activity (e.g., riding a bicycle), or during skillful absorbed coping (e.g., dancing) reflective activity is not always required and may be completely absent (and in some cases detrimental). These situations display pre-reflective aspects of the sense of agency. The reflective aspect is manifested in avowed volition, control, and intention. By contrast, actions that defy reflective intentions, such as crying in public unwillingly, phobic reactions to harmless situations, or addictions, emerge pre-reflectively. The lack of alignment between explicit intention and action is a consequence of conflicting norms and affectivities, tensions that remain unsolved. Voluntary (reflective) processes are not always able to avoid, delay, or control the accumulation of tension. These complex phenomena are usually collapsed into the binary distinction between willful/accidental, with important consequences for how we assign blame and social responsibility (Kirmayer and Gómez-Carrillo, 2019).

The distinction between reflective and pre-reflective intentionality offers more flexibility than dichotomies such as wilful/accidental or conscious/non-conscious. Pre-reflective activity is not hidden or inaccessible, nor are its manifestations in conflict or contradiction with the presence of reflective activity. The relation between these concepts is not so much one of logical opposition, but more like that of figure and background; complex and asymmetrical. Unlike reflective activity, which may withdraw in absorbed coping, pre-reflective activity never completely stops. A mathematician solving an abstract problem is a paradigmatic example of reflective activity, employing symbolic representations, and reasoning. But these processes are enabled by pre-reflective bodily intentionality, from intuitions and habits to adjusting the working environment to fit the task. Moreover, if at some point she stops working (reflectively) on the problem, pre-reflective processes may continue in the shadow, and sometimes, trigger intuitive insights. In contrast, an expert dancer that completely silences reflectivity during practice, or an expert rock-climber facing a well-known wall, are examples where pre-reflective activity dominates. Agency gets immersed in pre-reflective processes. However, absorption in an activity (in the absence of reflectivity) does not mean losing control over one's actions. There is an embodied intentionality at play. There are acts, dispositions, forms of bodily responsiveness, “intentional arcs” such as adjusting the body's relation to the world to attain maximal grip (Merleau-Ponty, 2012) that are not directed by conscious or explicit goals (Dreyfus, 2002; Custers and Aarts, 2010). Although these activities are dominated by pre-reflective processes, reflective activity may reappear and contribute to these performances. A rock climber will take a pause and consider the wall rising in front of her before moving again in absorbed “mode.” Reflective processes are very present at the initial stages of learning a skill and diminish with increased expertise (Dreyfus, 2002).

Expectation explanations of placebo effects rely primarily on conscious mental activity or non-conscious processes performing reflective-like functions (e.g., inference, prediction). The pre-reflective dimension is hardly considered. In contrast, Frenkel (2008) suggests that placebo effects can be explained exclusively by pre-reflective activity, that is, involving only non-conceptual motor intentionality. His position finds support in studies that show no modulation of placebo effects with cognitive tasks (Buhle et al., 2012). However, there are studies showing modulation of placebo effects with cognitive abilities as well, at least according to some measures. Individuals with higher IQs or with less severe symptoms in populations suffering from Alzheimer's show more pronounced placebo effects (Benedetti et al., 2006; Curie et al., 2015). So while we must bring in pre-reflective processes into placebo explanations, we cannot do away with reflectivity altogether.

The need to avoid dichotomies is implicitly recognized by some researchers. For instance, Geers et al. (2019, p. 212) distinguish between “low-thought” and “high-thought” expectations “(‘I believe the pill will reduce my pain just because a doctor said it' vs. ‘I believe it because a doctor made me actively think about the compelling arguments presented’)” not as binary categories, but as distributed along a continuum. This proposal considers different levels of reflectivity, but still neglects pre-reflective activity. As “high-thought” expectations are more robust and durable, predict better placebo effects, and are more likely to affect behavior than “low-thought” expectations, the authors suggest that increasing the level of reflectivity is likely to facilitate placebo effects. However, excessive expectations can lead to disappointment, despair, and harmful responses when things do not evolve as expected. The active and diverse phenomena of hopes (Eaves et al., 2016), often difficult to disentangle from expectations (but not reducible to them), may play a balancing role to get partial benefits of expectations while minimizing risks (Kaptchuk, 2018).

In other situations, excessive reflectivity can lead to worries and anxiety, generating tension, seizing attention and resources, and curtailing pre-reflective processes. Placebo phenomena have been associated with the activation of self-healing mechanisms (e.g., Walach and Jonas, 2004), the modulation of the immune response (e.g., Evans, 2005; Pacheco-López et al., 2006), and reduction of anxiety (Petrovic et al., 2005). We propose that tension release (diminishing harmful reflective activity) and the modulation of pre-reflective activity, such as the (re)activation of previously blocked processes, are likely to underpin global effects such as the ones mentioned above—an interpretation compatible with affordances of healing (Frenkel, 2008) and with opportunities to “reprioritize tasks” in environments perceived as safer (Trimmer et al., 2013). In contrast to the passive picture, the patient is always already flowing in a set of physiological, cognitive, affective, and interactive engagements. These activities make a difference.

To see the explanatory possibilities afforded by the category of pre-reflective intentionality, consider that placebo analgesia might improve therapeutic outcome by altering the range and variety of movements performed involving the injured area, through bodily adjustments and accommodations, changes in attention, or interpretation of bodily sensations. These are processes akin to regenerating habits, adopting new bodily postures, and a different awareness of bodily possibilities and sensitivities. Fostering activity in pain situations is common in physiotherapy. We have already mentioned that walking therapy in cases of arterial disease increases pain-free walking distance (Mol, 2002), but it also enhances social life and personal narratives pre-reflectively (as a by-product not reflectively linked to the original pain nor to the explicit goal of the therapy), which, in turn, may lead to renewed bodily confidence and positive looping effect on the success of the therapy. We would be surprised if placebo interventions did not have a significant effect on pre-reflective bodily processes, but these are not systematically studied in this context.

The shift from the relatively static dichotomy between conscious/non-conscious states to a more nuanced, and dynamic distinction between reflective and pre-reflective activity provides a tool for moving from a mechanistic, relatively passive view with the patient receiving and responding to treatment, to a more relational and active conception. Accordingly, we can conjecture that placebo interventions do not by themselves cause placebo effects, but rather trigger them by intervening in the existing configuration of entwined active processes in the current situation of the agent. This proposal can account for the variability, non-specificity, and unpredictability of placebo effects. The explanatory burden is shifted from the placebo intervention as such to the relational context of processes that are already active and which a variety of different interventions could trigger, modulate, or perturb to similar effect.

Such a shift toward a more active relational perspective on forms of bodily intentionality may require novel experimental paradigms to assess the impact of placebo interventions on personal and social practices, habits, and other behavioral patterns. For instance, a relatively recent experimental paradigm that can be explained in terms of the dynamics of reflective and pre-reflective processes is that of open-label placebos (OLP; Kaptchuk et al., 2010), as we shall discuss in the next section. Patients are reflectively aware of the fact they are taking a placebo. But they are still encouraged to undergo the whole ritual of the intervention, which, as it progresses, must still be accommodated in a bodily fashion, and these socially induced pre-reflective adjustments may lead to improvements in a variety of conditions (Charlesworth et al., 2017).

## The Therapeutic Encounter as Participatory Sense-Making

There is evidence to suggest that a strong and positive therapeutic relationship improves therapeutic outcomes in diverse fields, from physiotherapy (Kinney et al., 2020) to psychiatry (Martin et al., 2000). Similarly, an empathic interaction with a competent therapist tends to elicit placebo effects (Kaptchuk et al., 2008; Howe et al., 2017). At the same time, harmful nocebo responses can arise from unsympathetic, perfunctory therapeutic encounters (e.g., Benedetti et al., 2007). Any diagnostic act involves a social encounter that has the potential to influence the therapeutic outcome (Kirmayer, 1994), both reflectively and pre-reflectively. Understanding why can benefit clinical practice.

In their lived experiences, the patient and the clinician or therapist approach the meaning of illness from different perspectives. The patient might be confronted with the body as an object, even as an alien object that generates bodily doubt (Carel, 2013). However, the body-as-object that she perceives is qualitatively different from the body-as-object perceived by the clinician (Toombs, 1987). Too often, the role of the clinician is reduced to extracting a known disease from the complex illness experience of the patient, sometimes attending only to the patient's body while neglecting or downplaying the patient's agency and experience. But, there is a lived experience that must be taken into account for this encounter to be successful. The patient is often suffering, with high doses of uncertainty and worry, and seeking help, meaning, and change. By contrast, the practitioner is supposed to have knowledge and skills, although she also needs to deal with uncertainty, time constraints, and expectations (from the patient and the institution) that generate pressures to act. These two separate positions might facilitate a passive role in the patient and an active paternalistic one in the practitioner. From an enactive viewpoint, these tendencies must be overcome for successful participatory interaction to fully develop (see De Jaegher, 2019). Meaning from these two distinct positions must find some common ground to develop a shared world that fosters healing (Toombs, 1987).

The outcome of a therapeutic encounter depends on what enactivists call participatory sense-making. Participatory sense-making occurs when a social encounter develops its autonomous dynamics while preserving the autonomy of the participants (De Jaegher and Di Paolo, 2007; De Jaegher, 2018). That interaction dynamics can take a life of their own is illustrated by the simple example of two people walking in opposite directions in a narrow corridor, who find themselves locked in a temporary dance of symmetric sideway movements in their attempt to move past each other. They remain in interaction thanks to their efforts to get out of it. Participants in a social interaction undergo phases of coordination at multiple levels, from synchronization of neural and other physiological variables such as heart rate to joint action and perception to the regulation of interpersonal distance, turn-taking, conversational tone and topic, gestures, and so on (e.g., Dale et al., 2013). Meaning is jointly created by navigating situations of coordination and recovery from coordination breakdowns. Individual sense-making, i.e., the way each participant regulates intentions, perceptions, actions, emotions, etc. is literally modulated, sometimes enabled, and even co-constituted, by the sense-making of other participants *and* by the dynamics of the interactive encounter (De Jaegher et al., 2010); hence *participatory* sense-making. This means that other participants are not merely sources of imperfect social information to be complemented by individual inferential or simulation processes. Both individual and interactive autonomies are key for this process to occur.

Participatory sense-making is less likely to occur when rigid or orchestrated patterns curtail participation. Crucially, this process does not reduce to individual skills, intentions, or actions, even though it depends on them. It depends also on the concrete dynamics of the interactive encounter. Being sensitive to both patient and practitioner, there is a third interactive element not entirely controlled by either. Paternalistic attitudes by practitioners not only hinder patients' autonomy but are also an obstacle to the autonomy of the interaction. At the same time, pressure and attempts to control the situation by patients can also produce breakdowns that negatively affect therapeutic outcomes. Most studies in placebo research focus on individual subjective states in patients—e.g., personality traits, motivational goals (Geers et al., 2005; Corsi and Colloca, 2017)—or practitioners—e.g., empathy, competence (Kaptchuk et al., 2008; Howe et al., 2017)—and their attitudes and beliefs (Darlow et al., 2012). The interaction process as such has not been sufficiently investigated.

Evidence, however, suggests that interactive elements play an important role in placebo phenomena beyond the states or skills of individual participants. Kelley et al. (2009, p. 793) compared interactive patterns under augmented (warm and empathic) encounters eliciting larger placebo effects and limited (neutral) therapeutic relationships in acupuncture treatments of patients with Irritable Bowel Syndrome (IBS). The results revealed a large difference when participation is allowed to emerge in the augmented condition: the practitioner “facilitat[ing] patient's speech,” not “being distant, aloof (vs. responsive and affectively involved),” or allowing space for improvisation by not “exert[ing] control over the interaction.” Fostering shared meaning seems important as well: paying attention to patients' experience, treatment goals, or “current or recent life situation,” or the therapist providing explanations while avoiding “a teacher-like (didactic) manner.” As a result, patients showed less resistance to “examining thoughts, reactions, or motivations related to problems” in the augmented condition. These factors are evidence that a patient's genuine participation is encouraged. Interestingly, this study found that patient extraversion, agreeableness, and openness influenced placebo effects, but only in the augmented condition, suggesting that interactive factors can sometimes override the effect of positive social traits. This may explain the lack of consistent results when looking for placebo responders. Individual factors would seem to be relevant in that they can enable a rich process of participatory sense-making, but participation as such may still be thwarted for other reasons beyond the control of the participants.

Kelley et al. (2009) also found variability in therapeutic outcome even among therapists following the very same protocol (see also Johns et al., 2019). A protocol must be enacted; its basic description does not capture speech tones, body language, atmospheres, moods, receptivity, or prejudices. Performance matters (Thompson et al., 2009; Myers, 2010; Czerniak et al., 2016; Chen et al., 2019). Interactive elements can be shaped, but not entirely controlled. If they were, then participation, by definition, would be absent or very poor. Indeed, implicit influences are ubiquitous in social interactions (Greenwald and Lai, 2020), and they lead to double-blind procedures in RCTs. As blinding techniques attempt to remove the impact of these implicit interactive elements, it makes sense that RCTs cannot measure their influence on therapeutic outcomes.

Open-label placebos (OLP; Kaptchuk et al., 2010; Charlesworth et al., 2017) are a good example of the relevance of participatory sense-making in therapeutic encounters. Participants in these interventions must overcome the “non-sense” of taking a substance they know to lack any physiological effect. Uncertainty emerges and shared meaning must be generated from scratch (even against pre-existing knowledge). The active and purposeful performance of the practitioner facilitates this process by allowing the autonomy of the interaction to develop in a certain direction. Meaning is shaped by including strict instructions as in “real” treatments (e.g., “taking the placebo pills for 21 days is important,” Hoenemeyer et al., 2018, p. 3), by fostering an “open mind” toward possible unknown processes, by talking about a “novel mind-body treatment” (Carvalho et al., 2016, p. 2767), or by explaining the powerful influences of placebo effects, conditioning, attitudes, and faith on a treatment (Kaptchuk et al., 2010). When there is no such rationale, placebo effects are significantly reduced (Locher et al., 2017). In addition to the rationale, we suggest that interactive elements play a key role as well. Through participatory sense-making, tension is generated between existing beliefs and the proposed treatment. This tension opens up potentialities for change, increasing opportunities for individuation processes to be triggered. Uncertainty—together with imagination (Hardman and Ongaro, 2020)—are enhanced in a certain direction, shaping the shared meaning construction and concretely modulating hopes and expectations. Evidence “suggests that ‘expectations’ and even ‘cognitive information’ are not necessary for OLP efficacy” (Kaptchuk, 2018, p. 319). As a result of participation, certain pre-reflective processes are encouraged while reflective aspects are minimized or put on hold.

Participatory sense-making pervades all social interactions, not only that between patient and practitioner. The placebo by proxy hypothesis (Grelotti and Kaptchuk, 2011) proposes that the wider social environment, through the feelings and perceptions of clinicians, family members, and friends, also contributes to placebo effects. Studies show that placebo effects in children can be modulated by the expectations of their parents (Rheims et al., 2008; Weimer et al., 2013). We suggest that it is the situatedness of embodied agents in complex social environments, and the meaning generated in participation, that modulates placebo effects. A placebo intervention on a child can alter the behavior of her parents, and their augmented enthusiasm, more positive attitudes, or less worry and concern, can affect the child's pre-reflective bodily responses.

Participation is always embedded in cultural processes. We cannot assume that these are simply contextual factors. Almost every healing procedure is associated with a ritual aspect of social performance (Thompson et al., 2009), which is related to the uncertainty of the situation that is out of one's control, and the hope for relief through the intervention. Practices such as praying, which has been associated with placebo effects, owe their apparent healing capacities to previous culturally meaningful social encounters (Luhrmann, 2013) that shape uncertainty, facilitate imagination, and enhance hopes—also when they are practiced individually. Faith healing goes along similar lines. Likewise, biomedical procedures also involve ritual aspects that can be extremely powerful. Invasive interventions are usually performed in more severe conditions, require sophisticated technologies, and are carried out by specialized personnel. This could explain why, for instance, placebo surgery can elicit long-lasting benefits similar to “true” surgeries in diverse conditions (Jonas et al., 2015). Indeed, healing rituals alone—without associated “specific” procedures—have been performed for centuries, and they still play a role in many cultures (Kaptchuk, 2011). We understand rituals as triggering meaning responses and individuation processes, i.e., drastic changes in the set of tensions that are part of the embodied experience of the individual in her context. These individuation processes contribute to releasing or regulating part of the existing tensions and as a result, can lead to alleviating suffering.

The enactive perspective on participatory sense-making calls for widening the focus of placebo research to include the richness of culturally embedded social interactions as processes of participation, over and beyond the social skills of the participants. These processes are amenable to quantitative and qualitative research (Kelley et al., 2009; De Jaegher et al., 2010).

## Cultural Variability in Placebo, Health and Illness

Moving beyond dyadic interactions and taking into account social processes at different scales, the enactive approach provides elements to explain cultural variability in placebo, health, and illness (e.g., Moerman, 2000, 2002; Hutchinson and Moerman, 2018). To give an example of this variability, it has been shown that the color of a pill can modulate placebo responses, typically red having an effect of stimulation and blue of sedation (Jacobs and Nordan, 1979). An exception seems to be Italian men, who, at least at some point, showed a tendency to respond to blue-colored pills oppositely (blue is the color of the Italian national soccer team) (Cattaneo et al., 1970; Lucchelli et al., 1978).

Moerman (2002) notes that Chinese Americans that show certain combinations of disease and birth year that are “ill-fated” according to Chinese astrology die significatively earlier than non-Chinese Americans born in the same year (Phillips et al., 1993). The stronger their belief in astrology the earlier they die. The system of beliefs, social practices, and cultural narratives not only modulate the response to particular interventions—as in the ritual aspect of placebo procedures—but strongly transverse meaningful embodied experience. Although close to placebo and nocebo phenomena, these influences are not strictly placebo effects, as they lack a concrete intervention (i.e., a common trigger). As a result, they may be captured by the concept of cultural affordances, “the possibilities for interaction with a particular social context or eco-social niche from which an individual's experience, intentionality, and action-readiness emerge.” (Kirmayer and Gómez-Carrillo, 2019, p. 176). Complementing Frenkel (2008), they can be considered as “cultural affordances of illness,” which can give rise to “cultural nocebos” (the trigger could be the diagnosis of the disease or bodily sensations interpreted as symptoms of the disease).

Similarly, the medical and legal context seems to play a relevant role in the late whiplash syndrome. This condition involves a cluster of chronic symptoms (e.g., neck pain, headache, cognitive impairment) that are associated with rear-end car collisions, but its lack of existence in some non-Western countries, like Lithuania, awakened controversy (Schrader et al., 1996; but see Freeman et al., 1999). The general awareness of the syndrome by physicians and by the public, the possibility of obtaining financial compensation, the expectation of a severe injury (use of collar and stretcher after the accident), misattribution of medically unexplained symptoms, and attention (by oneself, by going to a practitioner frequently, or by friends and relatives) can contribute to this condition (Ferrari and Schrader, 2001). These explanations and those offered for placebo effects are remarkably similar and support our claim of the generality of meaningful embodied and situated interactions underlying placebo phenomena. “Experience is always preceded by and embedded in cultural systems of meanings and practices, which shape modes of attention and action, as well as interpretive frames and discursive practices that have causal effects as part of hierarchically nested loops linking social position, cognition and bodily processes.” (Kirmayer and Gómez-Carrillo, 2019, p. 172). In this view, placebo phenomena are just a subgroup of meaningful interactive experiences eliciting bodily responses, which are challenging to account for with current biomedical models.

## Enactive Conceptions of Health

To see, as enactivist do, the patient engaged in a series of mutually dependent and entangled processes at different scales, tallies fairly well with non-reductionist accounts of pathology and health. In contrast with the mechanistic tendencies in biomedicine, Goldstein (1954) conceived pathology as inherently relational, involving the whole organism in interaction with her sociomaterial environment. Pathology cannot be reduced to a symptom or dysfunction but depends on the subjective and objective assessments of available skills, practices, and capabilities for one's self-realization in a given context. According to Canguilhem (2012), external norms, based on statistics, cannot set the boundary between the normal and the pathological. Group to individual inferences are risky. The entangled dimensions of embodiment and multiple processes of individuation at play in each person are at the root of this difficulty. The pathological, then, is more related to the powers and potentialities for following/changing/incorporating/establishing a patient's own norms, than to the patient's position concerning the clinician's norm. For instance, people with autistic traits (Mercier et al., 2000), or impairments in motor control (Toro et al., 2020), can enjoy satisfactory lives and perceive themselves as healthy, despite of their deviation from standard bodies, capabilities, and ways of interacting common in Western societies (see De Jaegher, 2013).

Following Canguilhem, health depends on the relative capabilities for coping when confronted with novel or dangerous situations, or when breakdowns occur, i.e., on the ability to release or regulate tension generated in such cases. We have described living bodies as self-individuating processes that are continuously creating opportunities for further individuation. The available potentialities, however, require releasing or transforming tensions. If the processes of generating and releasing tensions cannot operate, tensions accumulate and other processes can attempt to compensate, with only partial or no success, and potentially causing other problems. As a result, bodily symptoms, dysfunctions, limitations, and suffering emerge as “positive” compensations rather than “negative” lacks (Merleau-Ponty, 2012).

The biopsychosocial model classically proposed by Engel (1977) (see also George and Engel, 1980), with its origin in Systems Theory, its emphasis on the whole person situated in her environment, and its critique to reductionist and dualist assumptions in biomedicine, also resonates with the enactive approach. However, it has been often applied in a fragmented manner (Stilwell and Harman, 2019), considering the “bio-psycho-social” realms as separate entities, and not paying enough attention to the interaction and entanglements of these domains.

As we have said, tensions can be created at each dimension of embodiment or through conflicting normativities between different dimensions. They can be generated, transformed, and released using reflective and pre-reflective processes, usually through a combination of both. A placebo intervention can trigger the regulation of tensions leading to the individuation of new meanings and a reconfiguration of the biopsychosocial situation. The positive influence of enhanced self-efficacy in therapy and the effects of over-attributing improvements to one's efforts show the importance of meaning and the sense of agency in regulating tension. Not only does an action matter, but so does its interpretation, the meaning ascribed to that action as part of one's skills and capabilities for interacting in a particular context.

Lived experience plays a key role in illness and also in the healing process (Toombs, 1987). We have shown the importance of interactive elements in the therapeutic encounter. The capacity for social relatedness in clinicians and therapists (and patients!) is crucial to be able to generate shared meaning and modulate hopes and expectations in such a way as to facilitate recovery.

The transformative role often attributed to illness (Lindsey, 1996; Carel, 2013) is easy to accommodate from the perspective of active embodied agency. The mechanistic body leads to a clear-cut distinction between health and illness. By contrast, studies sensitive to subjective experience interpret health and illness as a change in degree along a continuum (Toombs, 1988; Lindsey, 1996). A change that is not a mere loss or excess, but that solicits a reaction. Illness is perceived along with accumulated tension that cannot be managed, constraining the possibilities for (inter)action—as in patients with arterial disease and pain that limits mobility (Mol, 2002). This uncertain situation demands attention, action, and a search for meaning. In contrast, the healthy are interpreted as capable of creating opportunities, displaying options to open new potentialities, and coping adequately with the tensions generated.

The processes of tension creation and release help explain placebo effects also in “healthy” individuals. Tension is not exclusive of illness or disease, but ubiquitous in living bodies, and meaningful interactions are the main mechanism for regulating tension. Most studies of placebo phenomena in “healthy” subjects (e.g., Fillmore and Vogel-Sprott, 1992; Arntz and Claassens, 2004) can be interpreted as artificially generating tensions (e.g., nociceptive stimulus, “cognitive” task) and analyzing differences in their regulation following interventions (e.g., placebo analgesia, sham caffeine).

## Enactive Contributions to Placebo Research

We have presented an exposition of some central enactive ideas. Table 1 summarizes the various assumptions we have identified within traditional explanations of placebo and their corresponding contrasting assumptions in the enactive approach. It is time to take stock and summarize the main elements of our proposal and how they can broaden current explanations of placebo phenomena and inspire future research.

- **Non-dualism**. Enaction offers a non-dualistic theoretical articulation for explaining the continuity between life and mind, including the concepts of autonomy and sense-making, the minimal requirements for agency, and operational definitions of social interaction and participatory sense-making. The enactive approach entails neither reduction to biology, nor psychology, but expresses a relational-dynamical perspective that is not trapped within the orbit of traditional dichotomies such as subjective/objective, body/mind, individual/society, etc.
- **Entwined dimensions of embodiment**. The enactive conception of human bodies goes beyond passive, physiology-bound, and mechanistic perspectives. Bodies are active processes of ongoing individuation and cycles of regulation involving complex and entangled organic, sensorimotor, and social dimensions. The multiple cycles of regulation interact non-linearly and reflect the active history-in-the-making of organism and environment. None of these dimensions can by itself define or constitute a human body. One dimension may be more relevant in particular cases, but all of them participate in multiple constitutive relations. This non-universalist conception of embodiment helps make sense of the variability and non-specificity of placebo and the wide range of factors that modulate these effects.
- **A relational-processual interpretation of placebo**. Following Simondon's work, human bodies are constantly achieving individuation and regulating tensions within and between their dimensions of embodiment: tensions between internal processes, between sedimented structures and potentialities, between internal and external dynamics, between the individual and the collective, and so on. The useful distinction between the preindividual, i.e., the potentialities for individuation, and those factors that act as triggers can shift the strategy for explaining what placebo interventions do. Interventions do not by themselves initiate a causal chain that leads mechanistically to placebo responses, but trigger individuation events in already unfolding dynamical processes, helping break a deadlock, modulating the relative intensities of physiological, sensorimotor, and collective individuation processes. Other times they have no effect at all.
- **Pre-reflective and reflective aspects of agency**. Since cognition is manifested in the active regulation of the coupling between bodies and environment, mental states from the enactive perspective are never entirely private, though they may have different overt and non-overt components. The distinction between conscious and non-conscious processes is specious from this point of view, particularly when non-conscious activity is loaded with complex cognitive functions, such as predictions or inferences. We propose a more workable and phenomenologically defensible distinction, that between pre-reflective and reflective aspects of agency. More workable because it is easier to access empirically and more defensible because it foregrounds the agency of the patient. Tension release (diminishing harmful reflective activity) and the modulation of pre-reflective motor intentionality, for example, can help make sense of placebo interventions that induce anxiety-reduction (Petrovic et al., 2005), unblock self-healing mechanisms (e.g., Walach and Jonas, 2004), or modulate immune responses (e.g., Evans, 2005; Pacheco-López et al., 2006).
- **Participatory sense-making**. Social and cultural factors are acknowledged to modulate placebo effects. However, less emphasis has been put on the irreducibly relational processes of interaction and the conditions that affect the degree and kind of participation in these processes. Meaning does not arise solely as an individual response to an intervention but is jointly constructed in a process of participatory sense-making. This explains why healing rituals and participation-enhancing factors can positively modulate placebo effects, over and above the social skills of the individual participants.

**Table 1:** Comparison of underlying assumptions in classical explanations of placebo phenomena and the enactive approach.

**Classical explanations of placebo**	**Enactive approach**
Reductionism: factors affecting placebo effects are studied in isolation, assuming linear additivity among them	Non-reductionist framework based on dynamical systems in mutual interaction at multiple scales
Dualisms: “specific” vs. “non-specific” factors, physiology vs. psychology, known vs. knower, individual vs. society	Non-dualistic approach leading to life-mind continuity and deeply entangled organic, sensorimotor, and intersubjective dimensions of embodiment
Little attention to lived experience: subjective measures employed only to supplement objective measures	Lived experience as constitutive of life and mind, demanding first-person methodologies in combination with third-person approaches
Passivity: patients undergo treatments as machines responding to external perturbations in a lawful manner	Patients are agents actively regulating interactions with their (social) environment. Placebo interventions trigger individuation processes within a set of interrelated patterns of tensions that each individual enacts
Representationalism: cognition (usually separate from affect) as the manipulation of representations mediating between perception and action	Perception and action are completely intertwined, giving rise to agency and the (reflective and pre-reflective) sense of agency. Cognition—tightly linked to affectivity—emerges from these meaningful interactions with the environment
Individualism: relational and sociocultural factors play only contextual roles	The intersubjective domain is both enabling and constitutive of experience. Interaction dynamics are not fully exhausted by the sum of individual actions
Temporality: snapshot measures of variables on a short timescale. No focus on cross-scale correlations and dynamics of living bodies	Living bodies are interpreted as unfinished entities in an ongoing self-individuation process, full of tensions and potentialities, and deeply influenced by their history and previous experiences. Placebo interventions act as triggers within this configuration

As a complement to these contributions, we can specify some research guidelines that can help overcome the limitations of the theoretical assumptions described earlier. We suggest (1) expanding the investigation of first-personal experience, (2) systematically studying the interplay between pre-reflective (habits, self-efficacy, emotional dispositions, attentional patterns) and reflective (expectations, but also hopes, motivations, narratives, perceived self-efficacy, and interpretations associated with the placebo intervention and the healing process) aspects of agency in a dynamic manner, and (3) analyzing in-depth interactive experiences and participative processes in addition to individual social skills.

If we take the entwined dimensions of embodiment seriously, this calls for the combination of diverse methodologies (from first-, second-, and third-person perspectives) to assess the impact of placebo interventions on the way of life of patients and unveil hidden connections between bodily activity, behavioral patterns, and interpersonal processes. This can be challenging, but good examples exist. Despite the usefulness of self-report scales and questionnaires, the complexity of human experience, especially pain itself, cannot be reduced to a few numbers. Given the insights provided by a few phenomenological studies already performed on placebo (such as the work by Kelley et al., 2009), we find it advisable to include phenomenological methods to investigate both individual experiences of patients and practitioners, and interactive experiences in placebo research (e.g., Langdridge, 2007; Davidsen, 2013). We find especially useful the proposal for enactive-informed phenomenological methods (Stilwell and Harman, 2021).

These first-person phenomenological tools should be combined with traditional third-person data, as proposed by neurophenomenology (Lutz and Thompson, 2003) to inspire novel experimental paradigms in placebo investigations. The Multimodal Assessment model of Pain (MAP, Wideman et al., 2019) is a novel framework in pain research that emphasizes personal narratives while mixing quantitative and qualitative methods. Although it is quite individualistic and mainly focused on reflective activity, it is a relevant working example applicable to placebo research. By analyzing the phenomenological work by Kelley et al. (2009) in-depth, and by looking at the implementation details of the OLP experimental paradigm, we intend to highlight the potential of investigating interactive experiences in placebo research, including the pre-reflective aspects—see, for example, the role of the clinicians' facial expressions (Chen et al., 2019) or clinicians performance in placebo analgesia (Czerniak et al., 2016). For instance, it would be interesting to compare OLP procedures with different rationales and different performances of the clinicians while tracking the personal interpretation of the intervention in each participant. Such studies may shed light on the role that implicit influences, uncertainty, and imagination play in this experimental paradigm. Depending on the condition under investigation, empirical work on placebo could be complemented with questionnaires (to assess pain, self-efficacy, moods, anxiety) and interviews with different kinds of openness and structure to better grasp individual experiences and narratives. These could help unveil emotional variations and pre-reflective phenomena previously unnoticed (Stilwell and Harman, 2021) and allow a more detailed account of expectations and different kinds of hope (Eaves et al., 2016). Studies could also be complemented with diverse measures to track bodily activity associated with emotional experiences (e.g., heart rate variability, skin conductance) and eye-tracking methods to investigate attentional patterns, for instance, after an injury associated with pain and movement limitations.

In addition to this non-exhaustive list, techniques originating in the fields of anthropology and ethnography can offer valuable tools for analyzing the impact of placebo interventions on the way of life of patients (ethnomethodology, Hutchinson and Moerman, 2018; meta-ethnography, Hardman et al., 2020). These methodologies allow tracking a variety of looping effects with therapeutic potential, as illustrated by the meta-ethnographic work by Mol (2002) already discussed. This work compares the way of life of patients with arterial disease in the lower limbs undergoing either walking therapy—that reduces limitations associated with the disease and enhancing social life—or surgery—directly affecting vessels, but also patient's life through hospitalization, visits, and attention from relatives and the consequences of an invasive procedure. Investigating personal experiences, narratives and practices is challenging but can lead to a more complete picture. We think it is necessary to shed light on the variety of interrelated processes underlying physiological, psychological, and sociological domains, boost our understanding of the striking individual variability pervading placebo phenomena, and take advantage of the therapeutic potential placebo research contains to improve clinical practice.

## Discussion

The enactive contributions toward a relational-processual conception of placebo effects and their relation to similar proposals will no doubt deserve further elaboration. Our emphasis on lived experience coincides with ecological-phenomenological accounts (Frenkel, 2008) but while we also recognize the role of pre-reflective processes such as motor intentionality and affordances of healing, we do not think a full explanation of placebo phenomena can be constrained to those. Human agency is also manifested reflectively, in language, self-control, and social interaction, and so phenomena such as expectations can indeed be key for understanding some placebo effects. The difference from more traditional explanations is that expectations are not conceived as private mental states, but as dynamic processes of cognitive individuation involving the situated body in all its dimensions.

Ongaro and Kaptchuk (2019) have suggested that it is possible to bring together the active aspects of agency emphasized by enaction and expectation-based explanations of placebo via recent work on predictive processing (e.g., Clark, 2015; Allen and Friston, 2018). Apart from unresolved problems that are relevant to placebo research, such as the difficulties in accounting for affect-biased attention using predictive processing (Ransom et al., 2020), the similarities with the enactive approach are only superficial. Without entering into such a controversial topic, we can briefly mention that enaction emphasizes active historical processes at physiological, psychological, and social scales, which realize path-dependent trajectories and explain the human bodily and psychological difference and variability (Di Paolo et al., 2017, 2018). There is also an emphasis on the interpenetration between the dimensions of embodiment. In contrast to these two key enactive ideas, the mathematical framework of predictive coding strictly depends on the postulation of non-equilibrium steady states that downplay the influence of history and on the statistical compartmentalization of the organism (via Markov blankets) that may be applicable in some circumstances, but in general negates the entwinement of organic, sensorimotor, and social processes, and the interaction across multiple scales enactivists defend (see Di Paolo et al., 2021). Regarding placebo research, we find especially puzzling the explanation of OLP in terms of predictive processing provided by Ongaro and Kaptchuk (2019), as a result of the tension between contradictory information. OLP involves information that is certain and precise (placebos are inert) and information that is rather uncertain and imprecise pointing in the opposite direction (placebos may sometimes “work”). According to this framework, different sources of information are weighted depending on their precision, and thus the fact that placebos are inert should be weighted strongly. OLP is, at first sight, a case that challenges predictive processing. If it does not, it can only be because other intervening factors distort the role played by a certain and precise piece of conscious information, but the danger of this move is that predictive coding can then be made to explain anything in this way.

The enactive perspective foregrounds sense-making and so it strongly resonates with explanations based on meaning responses (Moerman and Jonas, 2002). But we are not limiting the relevant processes to conscious semantic constructions elicited by an intervention. There is a danger of putting too much emphasis on the mental side of things, neglecting both organic and social processes. Our emphasis on the entanglement and ongoing activity along the three dimensions of embodiment aims at preventing underscoring any one aspect in isolation. This is not the same as saying everything matters to the same degree for every case, but that the methodological requirements to frame an explanation for particular placebo effects must be established first using a framework in which no dimension is neglected by default.

In this work, we have focused on the enactive notions of bodies, sensorimotor agency, and participatory sense-making. The phenomenological, the existential, the affective, and the sociocultural dimension have not been analyzed in detail but they play a key role in placebo phenomena, and we hope to have shown that the enactive framework is in a position to offer tools to investigate and accommodate them.

To sum up, explanations of placebo phenomena remain elusive due to a series of epistemic problems. These include the background assumptions with which placebo effects are approached, the methods with which they are measured, and the paradox-inducing dualisms that adopt as a premise a gulf between terms that satisfactory explanations must bring together. Epistemic problems require epistemic solutions. Some of these solutions can be found in embodied cognitive science, particularly in the enactive approach, but also in related perspectives informed by phenomenology, ecological psychology, or anthropology. The investigation of placebo phenomena requires an epistemic shift toward seeing patients and practitioners as active social agents engaged in the regulation of tensions within and between intertwined dimensions of embodiment. This shift calls for objective measures to be expanded and also complemented with an investigation of processes of participation and relatedness. The resulting “engaged epistemology” (De Jaegher, 2019) allows us to make sense of the particularities of concrete interactive experiences.

What could count as an explanation of placebo effects if they are so context- and person-dependent? Are adequate descriptions the best we can do? The enactive approach provides a framework for generating explanations and new hypotheses, but not at the expense of abstractions that rely on a desire for one-size-fits-all single principles. Phenomenological methods (Stilwell and Harman, 2021), ethnomethodology (Hutchinson and Moerman, 2018), meta-ethnographic techniques (Hardman et al., 2020), and further developments in embodied and dynamical systems approaches in cognitive science will also be necessary for the understanding of meaningful interactions in a relational and situated manner, both within the therapeutic context and beyond. Results and methodologies from diverse fields (including first-, second-, and third-person approaches) must be combined, looking at embodied activity in performing agents at different scales and dimensions, and taking into account subpersonal, personal, and collective processes. Such an effort will encourage a fruitful dialectic between personal particularities and scientific generalization improving our understanding of placebo phenomena, health, and human embodied experience.

## Data Availability Statement

The original contributions presented in the study are included in the article/supplementary material, further inquiries can be directed to the corresponding author/s.

## Author Contributions

IA and ED participated equally in the conception, execution, and writing of this article.

## Conflict of Interest

The authors declare that the research was conducted in the absence of any commercial or financial relationships that could be construed as a potential conflict of interest.

## Footnotes

^1^There are different versions of E-approaches, sometimes adding the ecological or the emotional dimensions to the list of Es (e.g., Stilwell and Harman, 2019).

^2^For reasons of space, we barely touch on some fundamental aspects of Simondon's philosophy of individuation. For introductions to his work, see Chabot (2013) and Scott (2014).
